# Supplementary material for: Characterization of brain‐derived extracellular vesicle lipids in Alzheimer's disease
Source: J Extracell Vesicles. 2021 May 11;10(7):e12089. doi: 10.1002/jev2.12089 (PMC8111496; doi:10.1002/jev2.12089)
Supplement: Supplementary file 1 — Supporting information. [file JEV2-10-e12089-s002.docx]

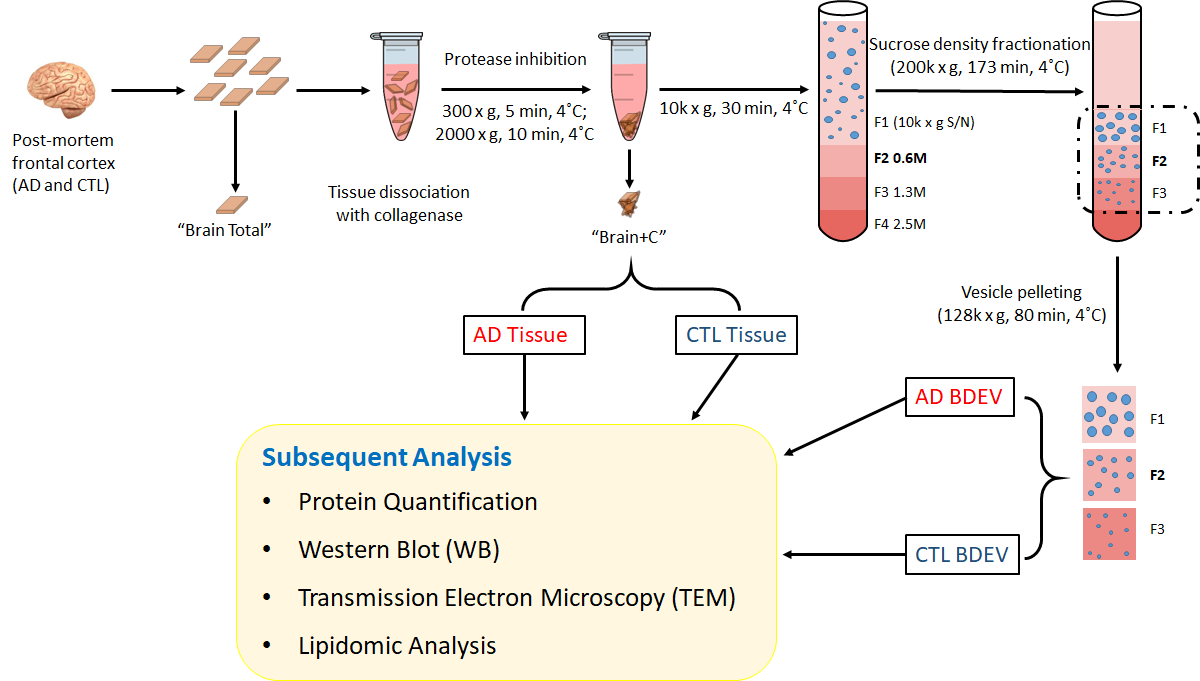


**Supplemental Figure 1. Summary of the experimental workflow for human post-mortem frontal cortex BDEV isolation and analysis.** Fresh frozen frontal cortex AD and CTL tissue samples (n=8 each) were sliced on ice. A small section of each was collected as a sample control (“Brain Total”). The remaining sections were dissociated with 50 U/mL of collagenase type 3 in DPBS at 25°C for a total of 20 min with shaking, followed by addition of protease and phosphatase inhibitors and EDTA. The dissociated tissue was spun at 300 x g for 5 min at 4°C (the resulting pellet was referred to as “Brain+C”). The supernatant was spun at 2,000 x g for 5 min at 4°C, followed by a 10,000 x g spin for 30 min at 4°C. The extracellular vesicle-containing supernatant was overlaid on a sucrose gradient and ultracentrifuged at 200,000 x g (avg) for 173 min at 4°C using a SW40 rotor (Beckman). The vesicles were separated into individual fractions based on density, then each fraction (F1, F2 and F3) was collected and transferred to individual ultracentrifuge tubes then pelleted at 128,000 x g (avg) for 80 min at 4°C using a F37L-8X100 rotor. “Brain Total”, “Brain+C” and vesicle-enriched fractions (F1, F2 and F3) were subjected to protein analysis. Fraction 2 (F2) was subjected to size and morphology validation by transmission electron microscopy (TEM). CTL = control, AD = Alzheimer’s disease, BDEV = brain derived extracellular vesicles.


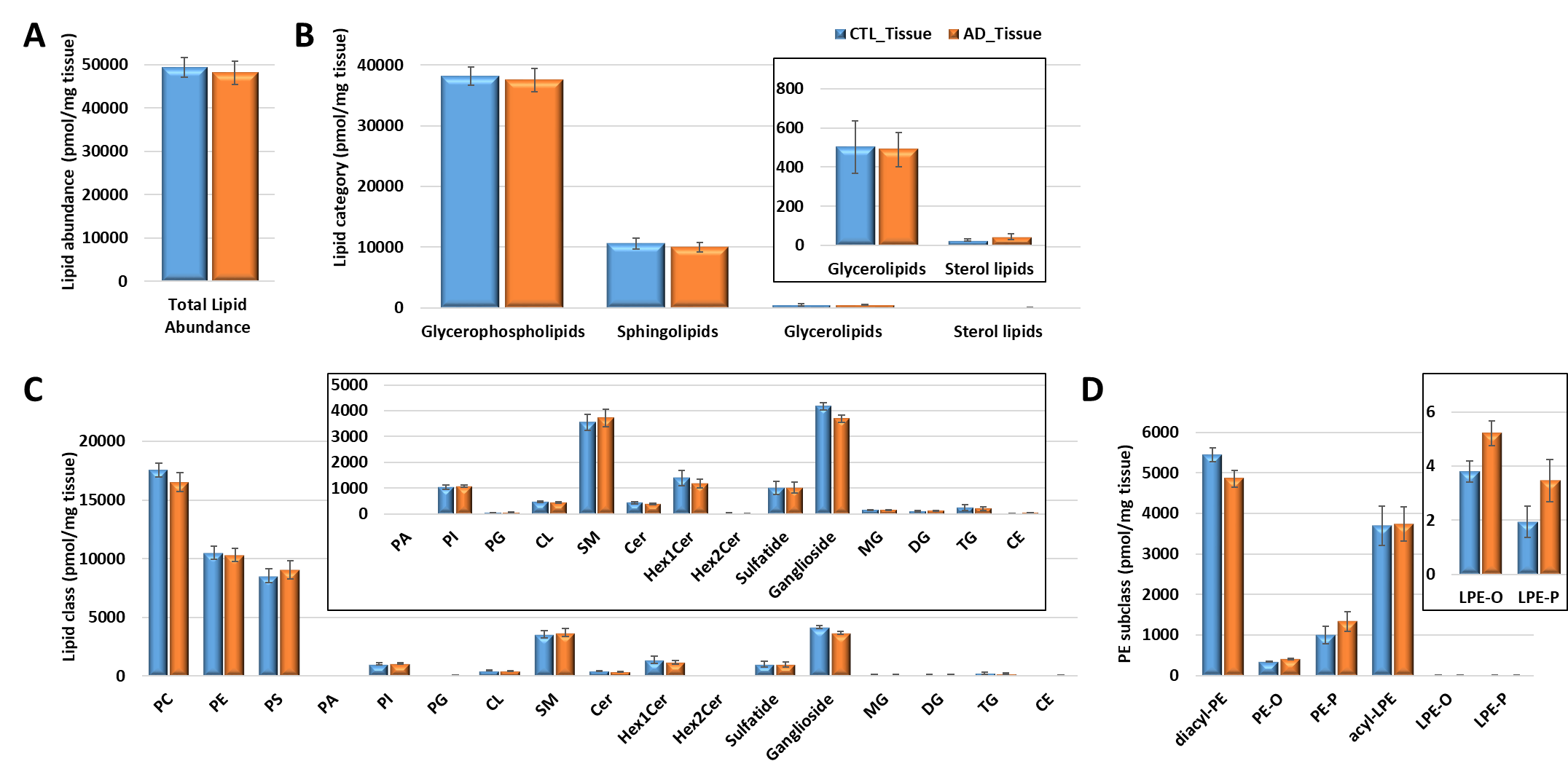


**Supplemental Figure 2. Comparison of lipid abundance (pmol/mg tissue) differences in frontal cortex tissue from control versus Alzheimer’s disease . (A) Total lipid abundance**. **(B) Total lipid abundance at the lipid category level.** Four lipid categories, covering glycerophospholipids (GP), sphingolipids (SP), glycerolipids (GL) and sterol lipids (ST) were included in this study. The inset shows the low abundant GL and ST categories for clarity. **(C) Total lipid abundance at the lipid class level.** A total of 17 lipid classes were identified in this study. The inset shows the low abundant PA, PI, PG, CL, SM, Cer, Hex1Cer, Hex2Cer, sulfatide, ganglioside, MG, DG, TG and CE classes for clarity. **(D) Total lipid abundance at the PE subclass level.** The inset shows the low abundant LPE-O and LPE-P for clarity. Data represent the average lipid abundance (pmol/mg tissue) ± standard error of the mean. Statistical significance was determined by ANOVA followed by Sidak’s multiple comparison test, with multiplicity adjusted p value < 0.01. CTL = control, AD = Alzheimer’s disease. N = 8 AD subjects and N = 8 CTL subjects.

**
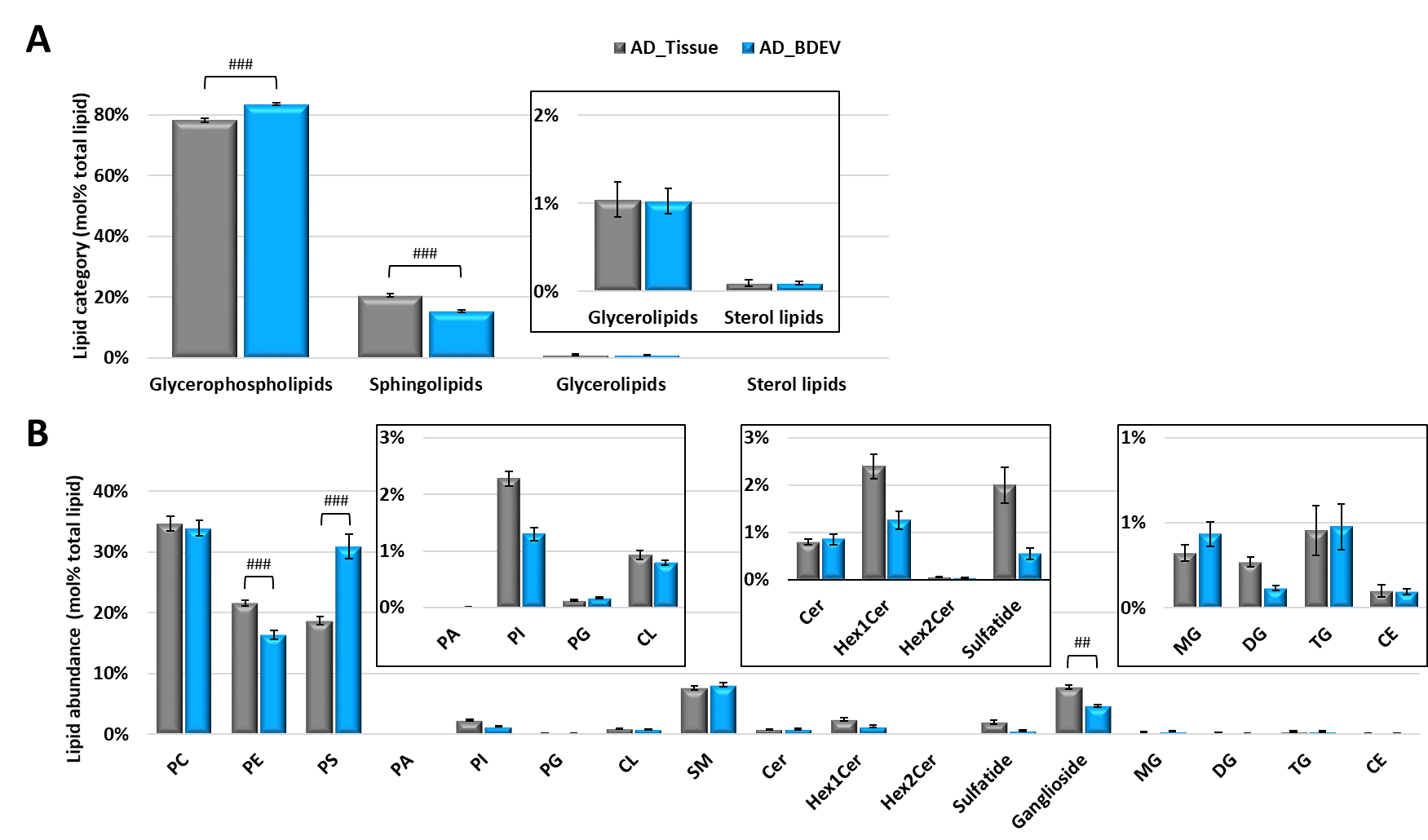
Supplemental Figure 3.** **Comparison of total lipid abundance differences between frontal cortex and the BDEVs in this tissue from Alzheimer’s disease subjects. (A) Mol% total lipid abundance distributions at the lipid category level**. Four lipid categories, covering glycerophospholipids (GPs), sphingolipids (SPs), glycerolipids (GLs) and sterol lipids (STs) were included in this study. The inset shows the low abundant GL and ST categories for clarity. **(B)** **Mol% total lipid abundance distributions at the lipid class level**. A total of 17 lipid classes were identified in this study. The inset shows the low abundant PA, PI, PG, CL, Cer, Hex1Cer, Hex2Cer, sulfatide, MG, DG, TG and CE classes for clarity. AD BDEVs were found to be significantly enriched in PS lipids, making up approx. 30% of the total lipid abundance, compared to AD tissue (approx. 19%). Ganglioside lipids were significantly downregulated in AD BDEV compared to AD tissue. Data represent the average mol% total lipid abundances ± standard error of the mean. Statistical significance was determined by ANOVA followed by Sidak’s multiple comparison test, with multiplicity adjusted p value < 0.01. ^#^ Adjusted p value < 0.01, ^##^ adjusted p value < 0.001, and ^###^ adjusted p value < 0.0001. AD = Alzheimer’s disease, BDEV = brain derived extracellular vesicles. N = 8 AD subjects.

**
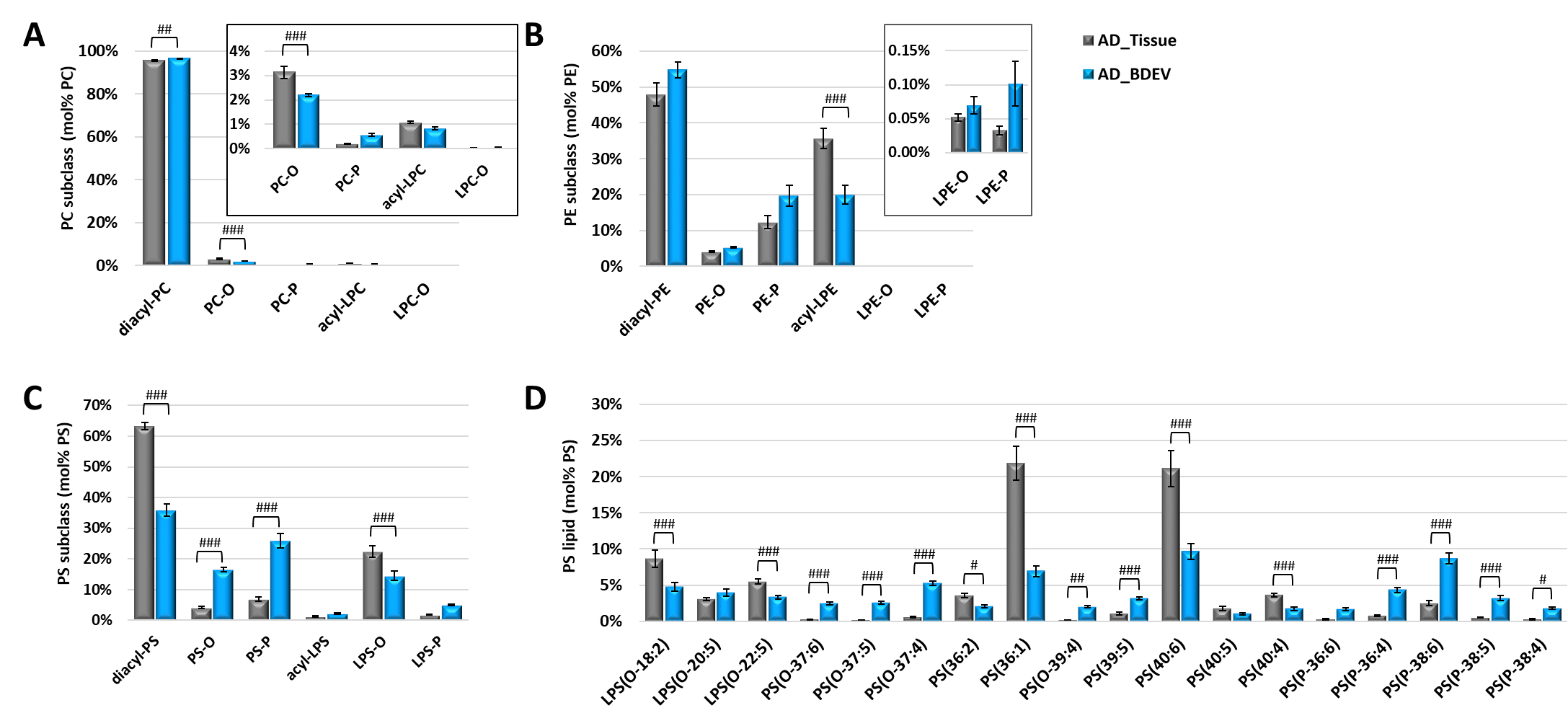
**

**Supplemental Figure 4.** **Comparison of PC, PE and PS lipid subclasses abundance and individual PS lipid molecules between Alzheimer’s disease tissue and BDEVs . (A) Mol% total PC lipid subclass abundance distributions**. **(B) Mol% total PE lipid subclass abundance distributions.** **(C) Mol% total PS lipid subclass abundance distributions**. **(D) Mol% total PS lipid abundance distributions of individual PS molecules.** Only the most abundant lipid molecules in PS lipid class are shown for clarity. Data represent the average mol% total lipid class abundance ± standard error of the mean. Statistical significance was determined by ANOVA followed by Sidak’s multiple comparison test, with multiplicity adjusted p value < 0.01. ^#^ Adjusted p value < 0.01, ^##^ adjusted p value < 0.001, and ^###^ adjusted p value < 0.0001. AD = Alzheimer’s disease, BDEV = brain derived extracellular vesicles. N = 8 AD subjects.


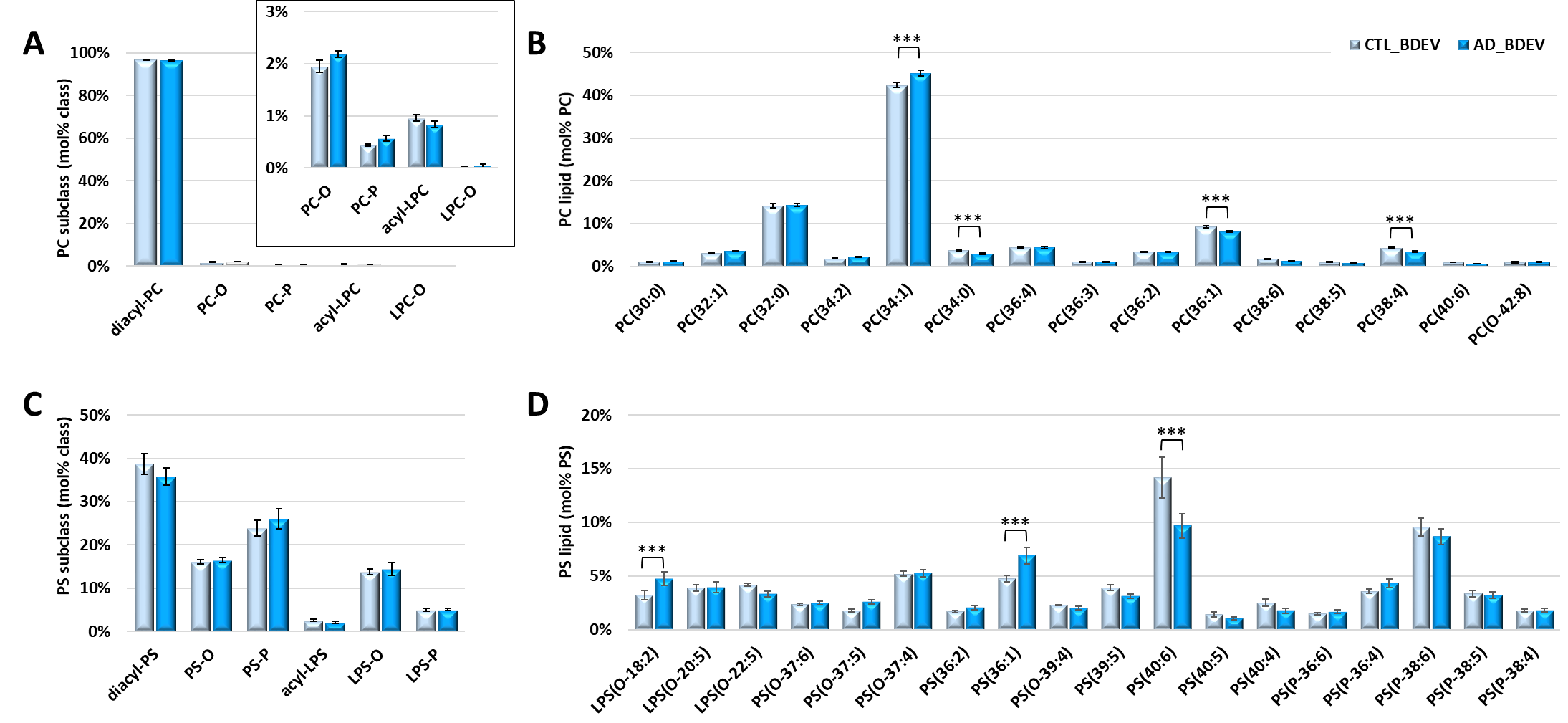


**Supplemental Figure 5. Comparison of total lipid abundance between control and Alzheimer’s disease BDEVs.** **(A) Mol% total PC lipid subclass abundance distributions**. No change was observed among PC subclasses between AD vs. CTL BDEV. The inset shows the low abundant PC-O, PC-P, acyl-LPC and LPC-O for clarity**. (B) Mol% total PC lipid abundance distributions of individual PC molecules**. Significant increase in PC (34:1) and corresponding decrease in PC(34:0), PC(36:1) and PC(38:4) were observed in AD vs. CTL BDEV. **(C) Mol% total PS lipid subclass abundance distributions**. **(D) Mol% total PS lipid abundance distributions of individual PS molecules.** LPS(O-18:2) and PS(36:1) were significantly upregulated in AD vs. CTL BDEV while PS(40:6) was significantly decreased. PS did not show distinct difference in ether species between AD and CTL. Only the most abundant lipid molecules in each lipid class are shown for clarity. Data represent the average mol% total lipid class abundance ± standard error of the mean. Statistical significance was determined by ANOVA followed by Sidak’s multiple comparison test, with multiplicity adjusted p value < 0.01. * Adjusted p value < 0.01, ** adjusted p value < 0.001, and *** adjusted p value < 0.0001. CTL = control, AD = Alzheimer’s disease, BDEV = brain derived extracellular vesicles. N = 8 AD subjects, N = 8 CTL subjects.


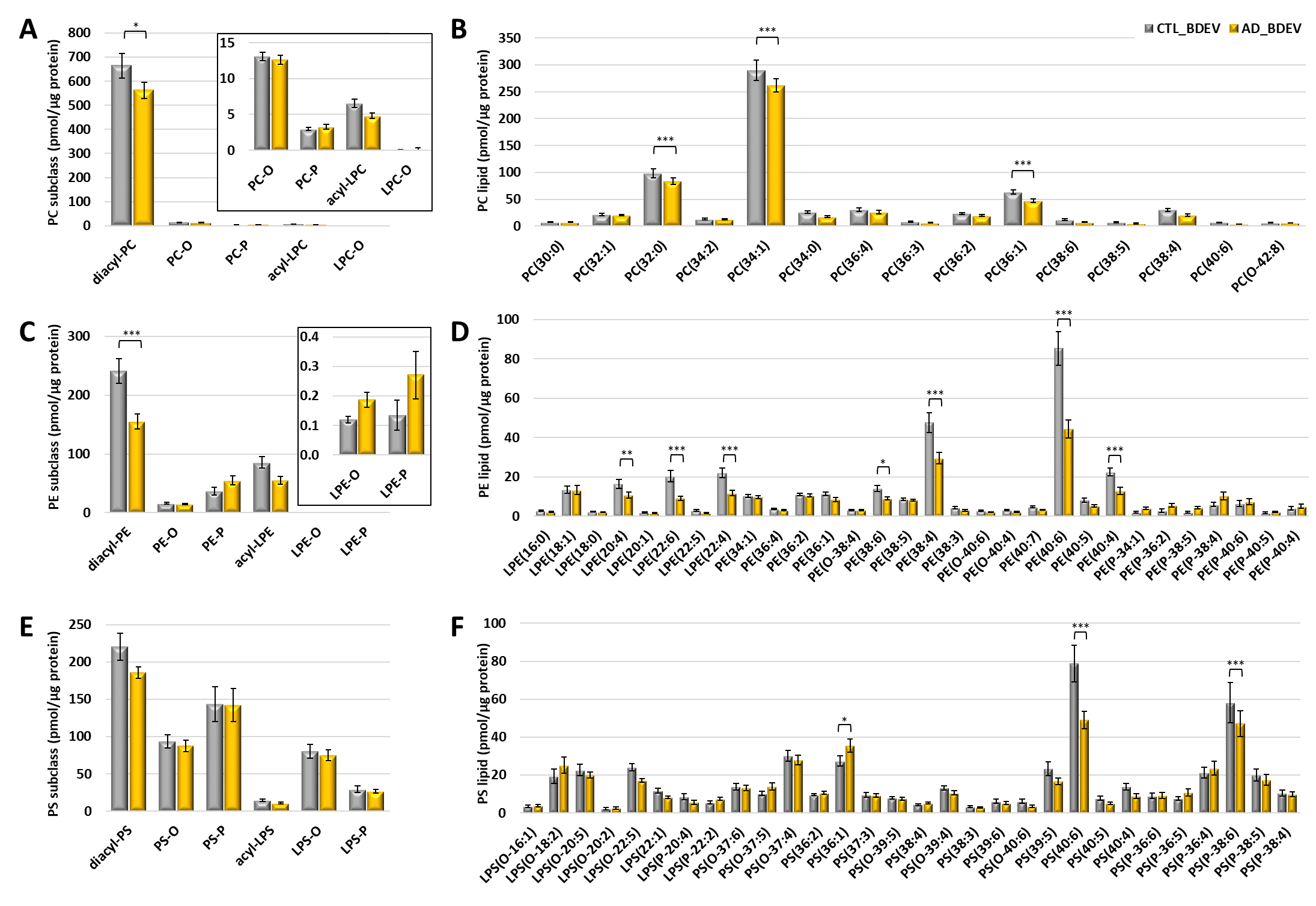


**Supplemental Figure 6. Lipid subclass abundance and individual lipid molecules (pmol/µg protein) in control and Alzheimer’s disease BDEVs. (A) Total PC subclass abundance.** Reduced total diacyl-PC abundance was observed in AD BDEV. **(B) Individual PC molecules.** Significant decreases in PC(32:0), PC(34:1), PC(36:1) and PC(38:4) were observed in AD BDEV. **(C) Total PE subclass abundance.** Reduced total diacyl-PE abundance was observed in AD BDEV. **(D) Individual PE molecules.** Polyunsaturated fatty acid (PUFA) containing PE molecules, including LPE(20:4), LPE(22:6), LPE(22:4), PE(38:6), PE(38:4), PE(40:6) and PE(40:4), were significantly decreased in AD BDEV. **(E) Total PS subclass abundance**. **(F) Individual PS molecules.** PS(36:1) was significantly upregulated while LPS(O-22:5), PS(40:6) and PS(P-38:6) were significantly decreased in AD BDEV. The insets show the low abundant species for clarity. Only the most abundant lipid molecules in each lipid class are shown. Data represent the average lipid abundance (pmol/µg protein) ± standard error of the mean. Statistical significance was determined by ANOVA followed by Sidak’s multiple comparison test, with multiplicity adjusted p value < 0.01. *Adjusted p value < 0.01, ** adjusted p value < 0.001, and *** adjusted p value < 0.0001. CTL = control, AD = Alzheimer’s disease, BDEV = brain derived extracellular vesicles. N = 8 AD subjects, N = 8 CTL subjects.


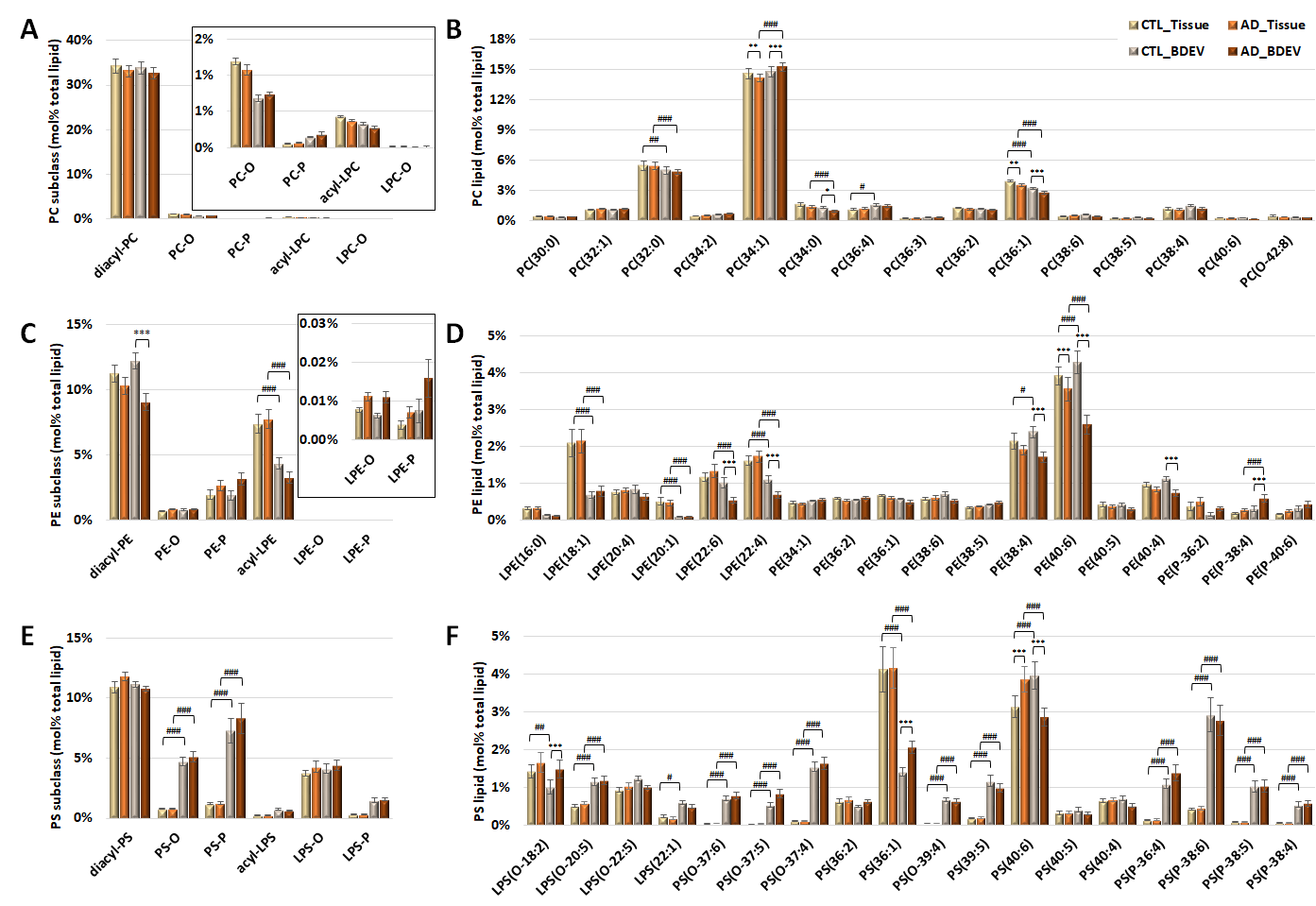


**Supplemental Figure 7. Comparison of PC, PE and PS lipid subclasses abundance and individual lipid molecules (mol% total lipid). (A) PC subclass abundance distributions (mol% total lipid).** The inset shows the low abundant PC-O, PC-P, acyl-LPC and LPC-O for clarity**. (B) Individual PC lipid abundance distributions (mol% total lipid).** PC(32:0), PC(34:0) and PC(36:1) were observed to be decreased, while PC(34:1) and PC(36:4) were observed to be increased in BDEV relative to tissue. Significant decrease in PC (34:1) and PC(36:1) were observed in AD vs. CTL tissue and significant decrease in PC(34:0), PC(36:1) and PC(38:4) were observed in AD vs. CTL BDEV. **(C) PE subclass abundance distributions (mol% total lipid)**. The inset shows the low abundant LPE-O and LPE-P. Acyl-LPE was decreased in BDEVs relative to tissue. Diacyl-PE was significantly decreased in AD BDEV compared to CTL BDEV. **(D) Individual PE lipid abundance distributions (mol% total lipid)**. An overall decrease in LPE (LPE(18:1), LPE(20:1), LPE(22:6) and LPE(22:4)) was observed in BDEV relative to tissue. Polyunsaturated fatty acid (PUFA) containing PE molecules, including LPE(22:4), PE(38:4), PE(40:6) and PE(40:4), were significantly decreased while PE(P-38:4) was significantly increased in AD BDEV relative to CTL BDEV. **(E) PS subclass abundance distributions (mol% total lipid)**. A significant enrichment of PS-O and PS-P was found in BDEV relative to tissue. **(F) Individual PS lipid abundance distributions (mol% total lipid).** An overall increase in ether PS species, including LPS(O-20:5), PS(O-37:6), PS(O-37:5), PS(O-37:4), PS(O-39:4), PS(O-39:5), PS(P-36:4), PS(P-38:6), PS(P-38:5) and PS(P-38:4), was observed in BDEV relative to tissue. LPS(O-18:2) was found decreased in CTL BDEV vs. tissue while the most abundant PS species, PS(36:1) was significantly decreased in BDEV relative to tissue. PS(40:6) was found increased in CTL BDEV vs. tissue but decreased in AD BDEV vs. tissue. LPS(O-18:2) and PS(36:1) were found to increase in AD vs. CTL BDEV. Interestingly, PS(40:6) was found to be increased in AD vs. CTL tissue but decreased in AD vs. CTL BDEV. Only the most abundant lipid molecules in each lipid class are shown for clarity. Data represent the average mol% total lipid abundance ± standard error of the mean. Statistical significance was determined by ANOVA followed by Sidak’s multiple comparison test, with multiplicity adjusted p value < 0.01. * represents comparisons between AD vs. CTL in either tissue or BDEV, # represents comparisons between tissue vs. BDEV in either AD or CTL subjects. * Adjusted p value < 0.01, ** adjusted p value < 0.001, *** adjusted p value < 0.0001, ^#^ adjustedp value < 0.01, ^##^ adjusted p value < 0.001, and ^###^ adjusted p value < 0.0001. CTL, control, AD, Alzheimer’s disease, BDEV, brain derived extracellular vesicles. N = 8 AD subjects and N = 8 CTL subjects.


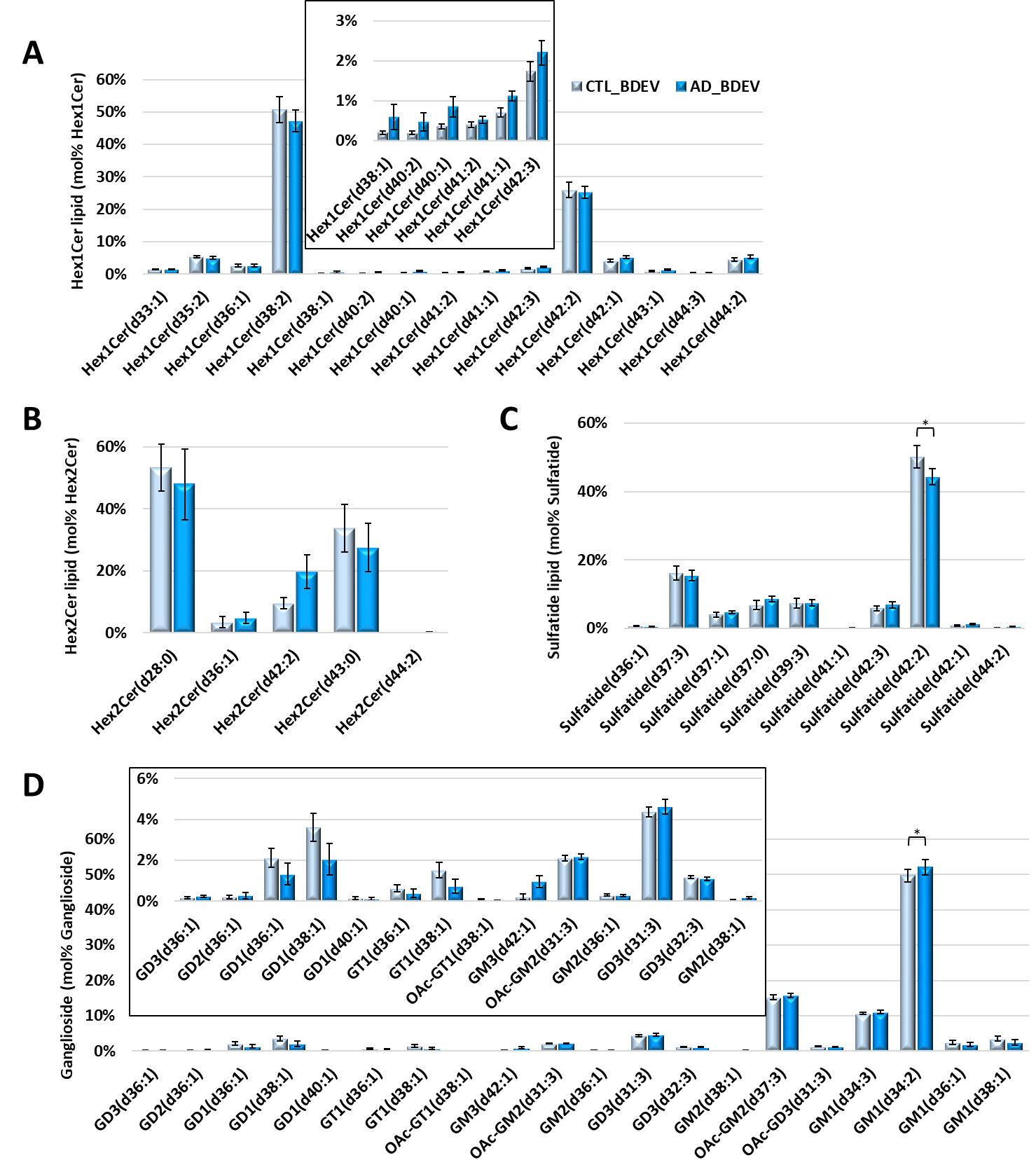


**Supplemental Figure 8. Hex1Cer, Hex2Cer, sulfatide and ganglioside individual lipid molecules (mol% class) in control and Alzheimer’s disease BDEVs. (A) Mol% total Hex1Cer lipid class abundance distributions.** **(B) Mol% total Hex2Cer lipid class abundance distributions**. **(C) Mol% total sulfatide lipid class abundance distributions**. Significant decrease in ST(42:2) was observed in AD BDEV. **(D) Mol% total ganglioside lipid class abundance distributions**. Only the most abundant lipid molecules are shown for clarity. Data represent the average mol% total lipid class abundance ± standard error of the mean. Statistical significance was determined by ANOVA followed by Sidak’s multiple comparison test, with multiplicity adjusted p value < 0.01. * Adjusted p value < 0.01. CTL = control, AD = Alzheimer’s disease, BDEV = brain derived extracellular vesicles. N = 8 AD subjects and N = 8 CTL subjects.


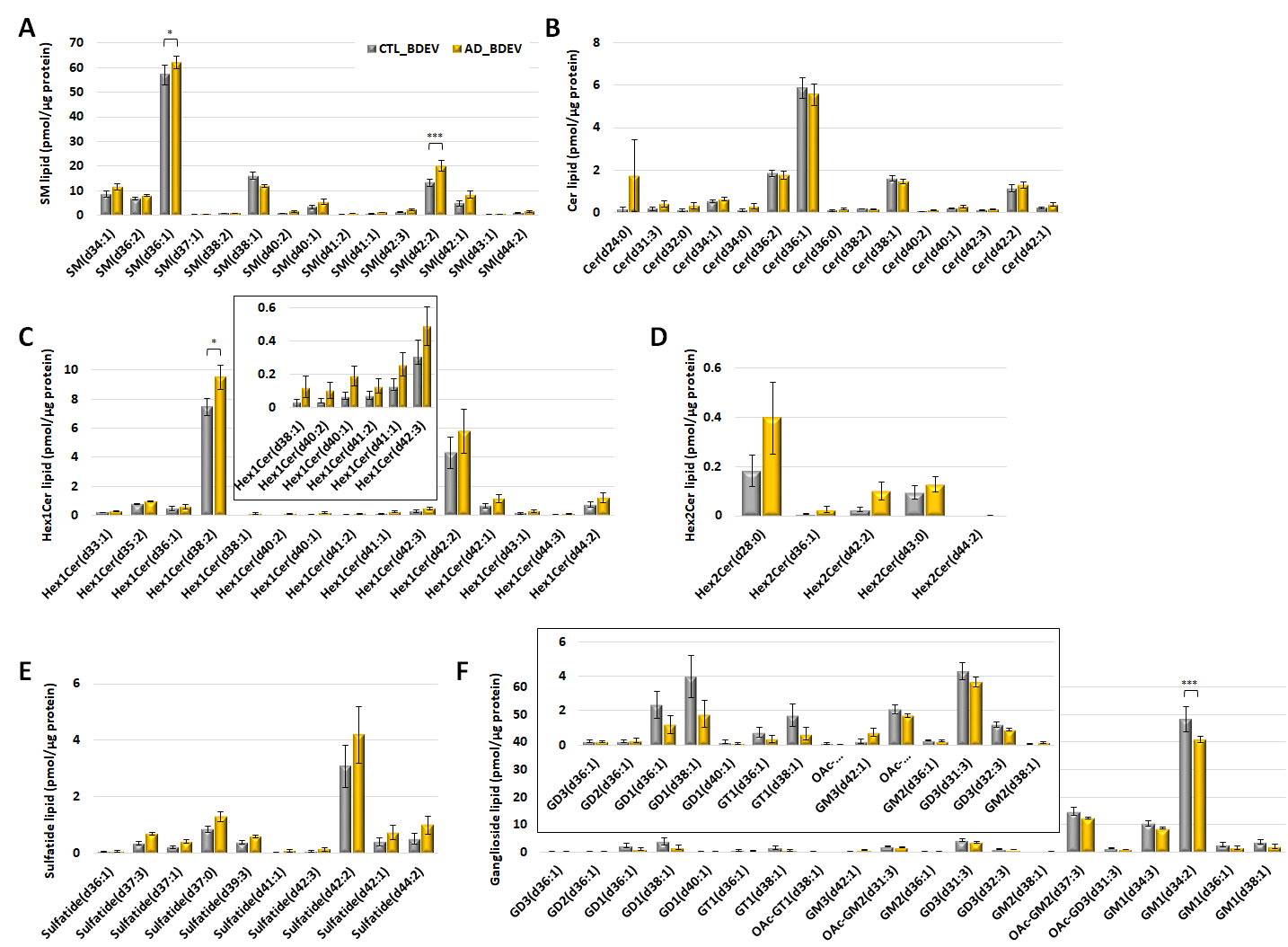
**Supplemental Figure 9. Sphingolipid molecules (pmol/ µg protein) in control and Alzheimer’s disease BDEVs.** **Individual (A) SM (B) Cer (C) Hex1Cer (D) Hex2Cer(E) sulfatide and (F) ganglioside molecules.** Only the most abundant lipid molecules in each lipid class are shown for clarity. Data represent the average lipid abundance (pmol/µg protein) ± standard error of the mean. Statistical significance was determined by ANOVA followed by Sidak’s multiple comparison test, with multiplicity adjusted p value < 0.01. * Adjusted p value < 0.01, ** adjusted p value < 0.001, *** adjusted p value < 0.0001. CTL = control, AD = Alzheimer’s disease, BDEV = brain derived extracellular vesicles. N = 8 AD subjects and N = 8 CTL subjects.


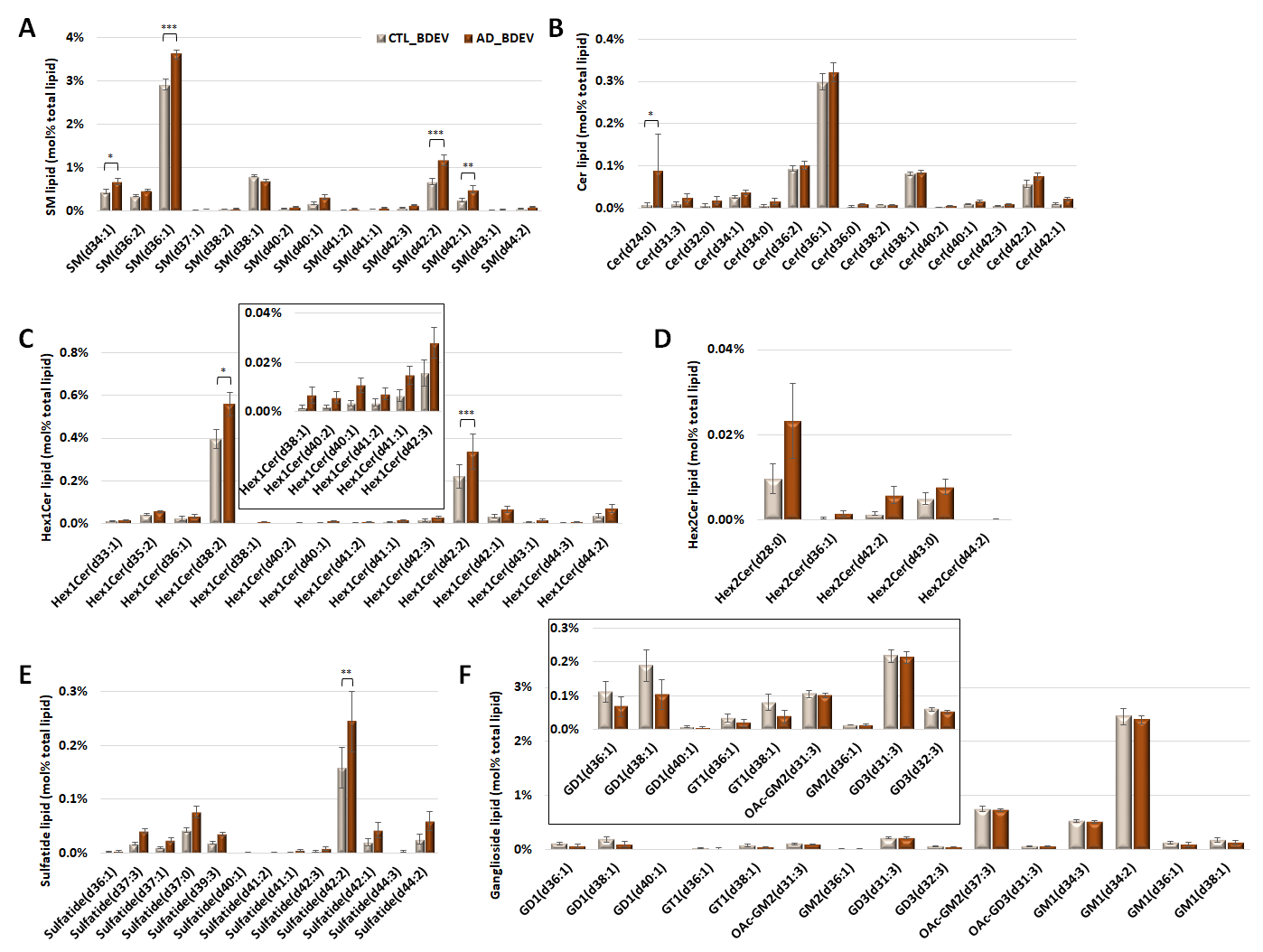


**Supplemental Figure 10. Individual sphingolipid molecules (mol% total lipid) in control and Alzheimer’s disease BDEVs. (A) Individual SM lipid abundance distributions**. Significant increases in SM(d34:1), SM(d36:1), SM(d42:2), predominantly the SM(d18:1_24:1) species, and SM(42:1), were observed in AD BDEV. **Individual (B) Cer** **(C) Hex1Cer (D) Hex2Cer (E) sulfatide and (F) ganglioside lipid abundance distributions**. Only the most abundant lipid molecules in each lipid class are shown for clarity. Data represent the average mol% total lipid abundance ± standard error of the mean. Statistical significance was determined by ANOVA followed by Sidak’s multiple comparison test, with multiplicity adjusted p value < 0.01. * Adjusted p value < 0.01, ** adjusted p value < 0.001, *** adjusted p value < 0.0001. CTL = control, AD = Alzheimer’s disease, BDEV = brain derived extracellular vesicles. N = 8 AD subjects and N = 8 CTL subjects.

**Supplemental Table 1. Subject case information**

| **Disease condition** | **Sex** | **Age**  **(yr)** | **Average age**  **(yr)** | **PMI**  **(hr)** | **Average PMI**  **(hr)** | **ApoE**  **genotype** |
| --- | --- | --- | --- | --- | --- | --- |
| AD | Male | 69 | 74.5 ± 7.0 | 13.5 | 23.3 ± 17.4 | E3/E4 |
|  |  | 72.1 |  | 13 |  | E3/E4 |
|  |  | 89.4 |  | 13 |  | E4/E4 |
|  |  | 76.5 |  | 26 |  | E4/E4 |
|  |  | 74.6 |  | 30 |  | E3/E4 |
|  |  | 76.4 |  | 19 |  | E4/E4 |
|  |  | 66.2 |  | 62.5 |  | E3/E3 |
|  |  | 71.4 |  | 9.5 |  | E3/E4 |
| CTL | Male | 78.3 | 73.5 ± 5.9 | 46 | 42 ± 16.3 | E3/E3 |
|  |  | 72.6 |  | 42.5 |  | E3/E3 |
|  |  | 75.6 |  | 46 |  | E3/E4 |
|  |  | 72.6 |  | 20.5 |  | E3/E3 |
|  |  | 66.9 |  | 21 |  | E3/E3 |
|  |  | 63.6 |  | 54.5 |  | E3/E3 |
|  |  | 77.5 |  | 69 |  | E3/E3 |
|  |  | 81 |  | 36.5 |  | E3/E4 |

Data represent individual subject information and the average value ± standard deviations (SD) in either AD or CTL subjects. AD, Alzheimer’s disease, CTL, control, PMI, post-mortem interval.

**Supplemental Table 2. Protein content of Fraction 2 (BDEV) in individual subject tissue samples.**

| **Disease condition** | **Fresh frozen tissue weight (mg)** | **Tissue protein (µg/mg tissue)** | **Average tissue protein (µg/mg tissue) ^a^** | **Total protein in Fraction 2 (BDEV, µg)** | **Vesicle yield ^b^** | **Average vesicle yield ^c^** | **Refractive index for Fraction 2 (BDEV) *** |
| --- | --- | --- | --- | --- | --- | --- | --- |
| AD | 2042 | 28.0 | 28.3 ± 5.3 | 294.5 | 0.1442 | 0.0785 ± 0.0342 | 1.3643 |
|  | 2038 | 31.5 |  | 222.1 | 0.1090 |  | 1.3644 |
|  | 2305 | 31.9 |  | 206.6 | 0.0897 |  | 1.3638 |
|  | 977 | 28.8 |  | 68.2 | 0.0698 |  | 1.3634 |
|  | 1219 | 35.7 |  | 76.3 | 0.0626 |  | 1.3652 |
|  | 1481 | 20.3 |  | 66.1 | 0.0446 |  | 1.3634 |
|  | 1131 | 21.1 |  | 52.0 | 0.0460 |  | 1.3641 |
|  | 1099 | 29.1 |  | 68.4 | 0.0623 |  | 1.3645 |
| CTL | 2283 | 43.1 | 36.5 ± 6.7 | 552.2 | 0.2419 | 0.1564 ± 0.0577 | 1.3627 |
|  | 1918 | 37.0 |  | 232.8 | 0.1214 |  | 1.3631 |
|  | 1988 | 32.6 |  | 485.5 | 0.2442 |  | 1.3636 |
|  | 2000 | 47.0 |  | 351.2 | 0.1756 |  | 1.3612 |
|  | 2015 | 38.1 |  | 236.9 | 0.1176 |  | 1.3614 |
|  | 1614 | 31.2 |  | 170.5 | 0.1056 |  | 1.3622 |
|  | 1213 | 37.4 |  | 134.2 | 0.1106 |  | 1.3646 |
|  | 994 | 25.6 |  | 133.2 | 0.1340 |  | 1.3632 |

^a^ Statistical difference of tissue protein (µg/mg tissue) between AD and CTL group was determined using unpaired student’s *t* test, p value = 0.0171. ^b^ Vesicle yield was determined from the protein content in vesicle fraction (F2, BDEV, in µg) as a function of the tissue weight (in mg) used for BDEV enrichment experiment. ^c^ Statistical difference of vesicle yield between AD and CTL group was determined using unpaired student’s *t* test, p value = 0.0054. *The refractive index in Fraction 2 (BDEV) corresponds to the density of approximately 1.08 g/ml (Vella *et al*., 2017). Data represent individual subject information and the average value ± standard deviations (SD) in either AD or CTL subjects. BDEV, brain-derived extracellular vesicles, AD, Alzheimer’s disease, CTL, control.
